# The Italian *Cilentana* goat breed: productive performances and economic perspectives of goat farming in marginal areas

**DOI:** 10.1007/s11250-022-03292-7

**Published:** 2022-09-15

**Authors:** Piera Iommelli, Lorenzo Infascelli, Raffaella Tudisco, Fabian Capitanio

**Affiliations:** Department of Veterinary Medicine and Animal Production, University of Naples Federico II, 80100 Naples, Italy

**Keywords:** Cilentana goat, Cheese, Kid meat, CAP post 2022, Farmer’s survival

## Abstract

In the internal areas of Cilento, province of Salerno (Campania), the Cilentana, a small native goat breed population, is reared quite widespread, due to its dual-purpose attitude and to its typical productions. The extensive livestock system adopted for this breed allows the use of otherwise abandoned territories and a sustainable farming capable of ensuring high-quality levels. In addition, Cilentana goat farming represents an important source of income for the local communities and also preserves the territory itself guaranteeing the protection of biodiversity and the conservation of local activities that have a historical tradition. The aim of this study is twofold: give an overview of Cilentana breed morphological and productive traits linked to the historical and gastronomic tradition of the area and emphasize the economic role of this breed in the perspective of the ongoing new Common Agricultural Policy (CAP) rules.

## Introduction

Goat farming in Italy is mainly grouped in the South and in the islands regions where farms are largely characterized by the use of local breeds. In the South, the goat has been present since very remote times. As a matter of fact, in Roman times, it occupied a role of considerable importance in the economic system: it was raised for the leather and meat as well as for the production of milk and cheeses more commonly consumed than that of cows (Rubino 1996).

Due to its nutritional properties, goat milk is considered an alternative to cow’s milk owing to the smaller size of the fat globules and consequently to its greater digestibility (Saini and Gill, 1991). Furthermore, 20% of the fatty acids in goat’s milk such as caproic acid, caprylic acid and capric acid are short chain, and as a consequence, they are rapidly digested (Jennes, 1980; Quarto et al., 2008). Positive properties of goat’s milk like the high content of taurine and conjugated linoleic acid (CLA), higher than other species’ milk, give to this breeding and its related productions an added value (Quarto et al., 2008; Delgadillo-Puga et al., 2020).

Another peculiar characteristic of goat milk fat is the presence of specific branched chain fatty acids, such as 4-methyoloctanoic and 4-ethyloctanoic, responsible for the typical ‘ircino’ (goatish) flavour of milk and cheeses (Quarto et al., 2008).

The Cilentana goat is a native breed of the Cilento area, to which it owes the origin of its name. According to ARAC data (2021), it consists of a population of about 3000 heads mainly reared with semi-extensive systems on pastures of the Cilento (Zullo et al., 2005). Cilento is a vast area, mainly characterized by mountainous landscapes, bordered to the North by the Sele River, to the East by the Vallo di Diano and to the South and West by the Tyrrhenian Sea. The vegetation of this area is the typical Mediterranean scrub, rich in mastic, myrtle and juniper trees. Along the hills, olive cultivation is widespread, while the inland areas are populated by deciduous broadleaf woods such as oaks, maples and chestnuts trees. In the sunnier areas, where the wood is less dense, there are polyphite meadows consisting mainly of grasses, some species of thistles and various aromatic plants (Indelli and Pratesi, 1994).

Although the natural beauty of the Cilento landscapes is undoubtedly very suggestive, it is considered ‘marginal’ due to the excessive slope of the territory and the high distance between the small inhabited centres which have always represented challenging obstacles for the technological progress of this area (Indelli and Pratesi, 1994).

The typical production of Cilentana goats is ‘Cacioricotta’, a traditional product of this area, characterized by its peculiar manufacturing technique. As a matter of fact, Cacioricotta is a cheese obtained by cooking milk at high temperature which guarantees the integration of globulins, albumin and whey proteins into the cheese paste, thus combining the compounds of cheese (‘Casu’) and ricotta, hence the name Cacioricotta (Regione Campania: Assessorato all’agricoltura).

Lately, political and scientific actions aimed at the protection of biodiversity have led to the enhancement of the native Cilentana goat (Quarto et al., 2005) with the purpose of protecting animal species barely present on the territory and of the preserving typical products culturally and traditionally linked to them (Sacarrão-Birrento and de Almeida, 2021).

As reported by Di Trana et al. (2015), local breeds, like Cilentana, appear to be the main component of animal farm biodiversity, thanks to their excellent adaptation to specific environmental conditions. From a wider perspective of sustainability which takes into consideration the social, economic and animal welfare aspects (Esposito et al. 2022), rearing these breeds preserves typical and traditional productions of marginal territories, giving to these breeding systems an added value. Referring to the economic perspectives, the above-mentioned launch of the new CAP programming, where ecosystem services will play a crucial role, aims at protecting and restoring the forest heritage and increasing biodiversity. Among the possible interventions, aids for controlled grazing could be envisaged: this is a method used in France and Spain and could also be tested in Italy (Caballero et al., 2011). Today, it represents a tool that can effectively help the prevention of fires where breeders have the opportunity to team up to collaborate, improve their extensive production systems and benefit from greater social and economic recognition of their activities. This could represent an ecosystem service provided by shepherds who operate in respect of the climate and biodiversity by reducing the risk of fires; at the same time, they would be rewarded thus improving their profitability. For this purpose, we will carry out a section on the new framework of CAP intervention since 2023.

The aim of this work is both to describe and characterize the Cilentana goat from a morphological and productive point of view and emphasize the economic perspectives of this breed in the new global scenario (e.g. Cap post 2023 and climate change). Concerning the first aspect, little is known about the origin of this breed, but many works have been done on the characteristics of its production (milk) (Tudisco et al., 2012, 2019a; Zicarelli et al., 2016) as well as on the chemical characteristics of available forage (Tudisco et al., 2019b) to properly estimate its nutritive value (Musco et al., 2016).

## Cilentana breed

Cilentana breed is an autochthonous goat population of the Campania region represented by three genetic types, the Tawny, the Black and the Grey, whose origins date back to crossbreeding between local populations, respectively, the ‘Derivata di Siria’, the ‘Garganica’ and the ‘Maltese’ breed (Noè et al., 2005). Little is known on the origin of this breed, but from the work of Fantazi et al. (2018), we can assume that it probably originates from the northern region of Africa since it shared more variants for the protein gene PRNP with Algerian breeds than other Italian breeds.

**Fig. 1:**
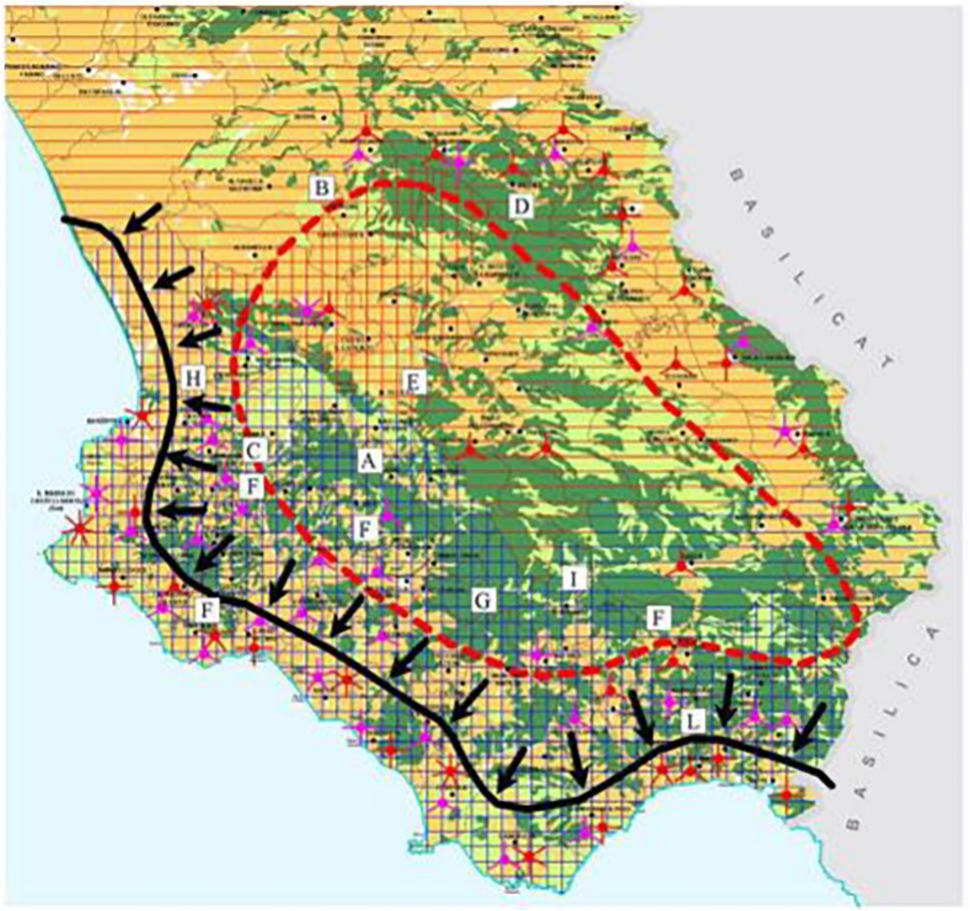
Cilento area. From Parco Nazionale del Cilento e Vallo di Diano (Piano del parco, relazione illustrativa). Letter L shows the area most interested by goat farms

It is mainly bred in the Cilento area, located in the provinces of Salerno, and specifically in the Cilento, Vallo di Diano and Alburni National Park (Fig. 1). In particular, according to ASSONAPA, there are 42 farms in the province of Salerno and only one farm in the province of Avellino.

The Park’s altitude ranges from sea level to the top of the Cervati Mountain, at 1898 m, thus including the inshore area characterized by a Mediterranean climate with an average temperature of 22–28 °C and the internal area where the effects of Mediterranean climate are mitigated and average temperatures of 15–20 °C: summers are less sweltering, but above all winters are cold (Di Novella et al., 2013; Aliberti et al., 2022).

Due to its rusticity, Cilentana goat is reared especially in small farms with extensive or semi-extensive systems and it is well adapted to the pedo-climatic conditions of the territory. The extensive or semi-extensive rearing system provides for the use of pasture, especially in the spring and summer seasons (Tudisco et al. 2014). In most cases, free ranging systems are adopted, where also state-owned pastures are used by the farmers. In the Cilento area, 9832 ha of surface is used in permanent meadows and pastures (Regione Campania, 2013). Shelter for the animals is always ensured and is particularly useful in the winter months when the animals are fed mostly hay as forage base and stay for longer in the pens. Cilentana breeding is aimed at the production of kids (for meat) and of typical cheeses.

In the last 30 years, its presence on the territory has been significantly reduced, and today, the animals are registered in the Registry of indigenous sheep and goats with limited circulation (active since 2002) (Noè et al., 2005). Indeed, the number of heads was about 5000 in the early 1970s on the territory of the Salerno province (Peretti et al. 2013). Its rearing guarantees the control of otherwise abandoned territories and allows the eco-sustainable use of available resources and the preservation of the historical, cultural and gastronomic heritage.

## Morphology

**Fig. 2:**
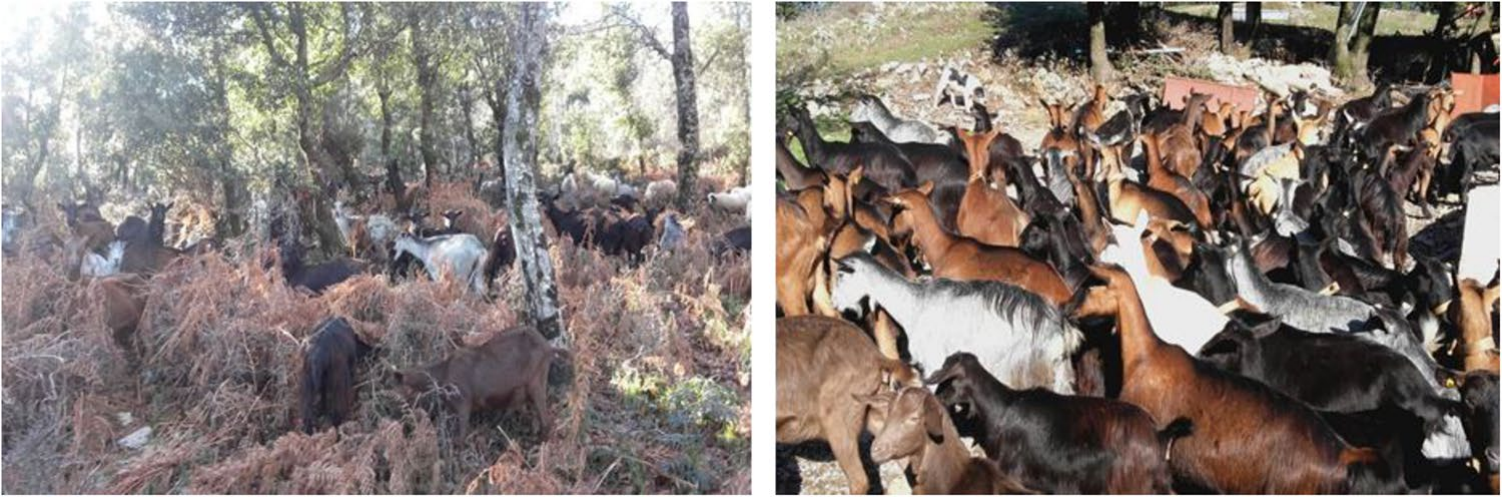
Cilentana goats of different genotypes

As above mentioned, there are three genetic types of Cilentana breed: the grey, the black and the tawny (Fig. 2). The Black Cilentana is the variety of Cilentana goat most present in Campania region, and indeed, 2671 heads are registered to ASSONAPA (Associazione Nazionale della Pastorizia); it is followed by the tawny type (189 heads) and by the grey one which is the least common type (78 heads) (Assonapa, 2022). The distinction among them does not concern only the coat, but also other important morphological and milk technological properties. Zullo et al. (2005) showed a comparison on αs1- casein allele of herds of goats composed by different genotypes. In particular, F genotype at the αs1- casein locus is the most frequent in the mixed herd and in the one composed only by the tawny type, while AF genotype was mainly present in the herd only composed by the black type. These differences are important on milk coagulation characteristics because they influence enzymatic phase duration, coagulation speed and curd consistency.

Cilentana goat is medium in size with a weight between 65 and 70 kg in males and 45 and 50 in females, even if the tawny type is lighter. The height at the withers is about 77 cm in males and 70 cm in females, although the black type is slightly taller (Istruzione agrarian online: www.agraria.org). The head is generally small, with horns present in half of the subjects and a beard present in most of them.

In all the three genotypes, the udder is firmly attached and is mainly of the bifid type, followed by the hypoglobose, semibifid and globose type (SAFE TGA, 2020).

**Fig. 3:**
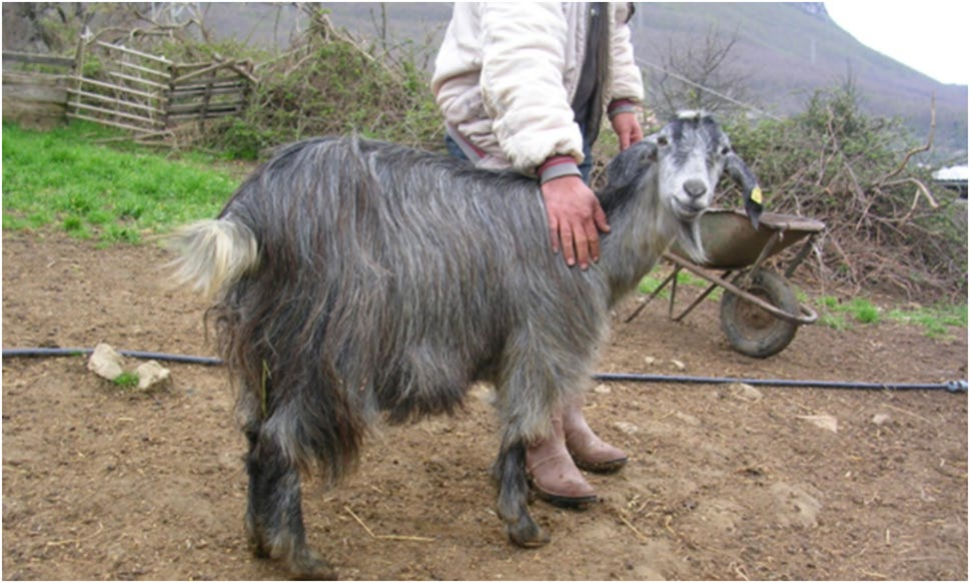
Grey Cilentana. From SAFE TGA

**Fig. 4:**
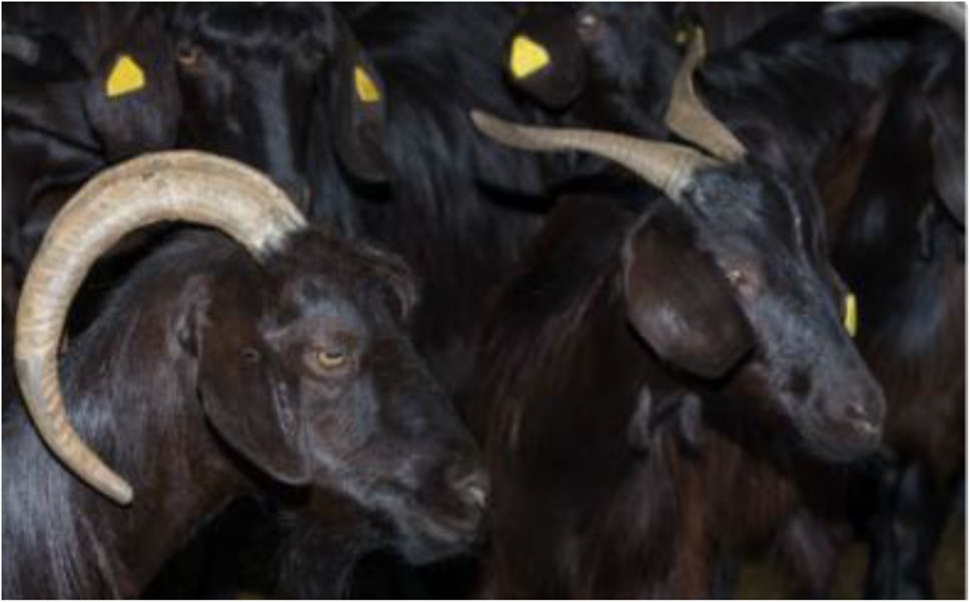
Black Cilentana. From SAFE TGA. On the left, the alpine type of horns; on the right, the garganica type

**Fig. 5:**
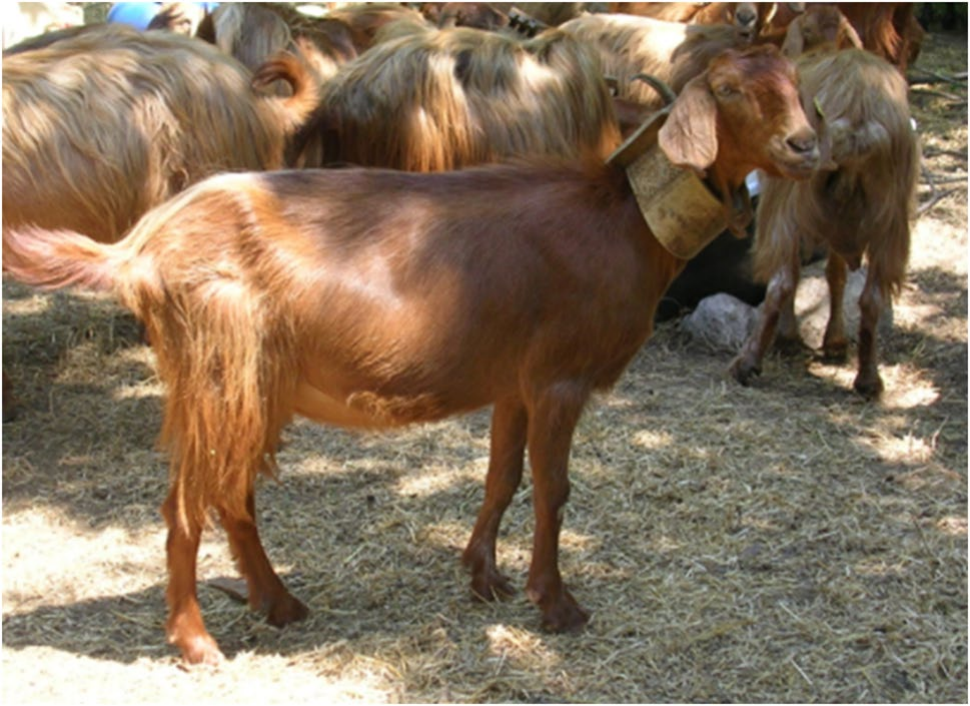
Tawny Cilentana. From SAFE TGA

The horns, generally less present in the tawny type (35%), can be of the alpine type (saber-shaped curved backwards), 20–30 cm long, or of the garganic type (vertically spiralling horns), 30–40 cm long (Peretti et al., 2013). The coat differs in the three genetic types. In the grey Cilentana, the coat is uniform grey generally with darker shoulders, neck and back, mainly long-haired in males and mixed in females (Fig. 3). In the black Cilentana, the coat is uniform black, with long hair in the males and mixed in the females; small patches of limited size can be present as well as small stockings but always accompanied by black nails (Fig. 4). In the tawny Cilentana, the coat is uniform tawny, long-haired in males and mixed in females; ventral white spots of limited size can be present as well as small stocking accompanied by dark claws and small percentages of white hair mixed with tawny (Fig. 5) (Noè et al., 2005).

## Feed requirements

Adopting the values reported by Rubino (1996) for all the local breed raised in the Mediterranean areas, Cilentana breed goats’ nutritional requirements were satisfied as demonstrated by several studies (Musco et al. 2022; Tudisco et al. 2010; Zicarelli et al. 2016). Many studies (D’Urso et al., 2008; Tudisco et al., 2014) have been carried out on the relationship between feeding and characterization of production (milk); the main diets considered for these animals are reported below, according to what are the rations mainly used by farmers.

When the climatic conditions are favourable, pasture is used as a forage base, generally in spring and summer. Pasture intake has been calculated by Rubino (1996) who reports 20 g/kg body weight (BW) as average pasture dry matter (DM) intake or 3.5% of live weight and considers the energy requirements for maintenance and milk production of 0.0365 UFL (feed unit for lactation)/kg metabolic weight (MW = BW0.75) and 0.41 UFL/kg fat-corrected milk (4% fat), respectively. Therefore, since body weight is around 50 kg, pasture intake is on average 1–1.5 kg of DM which is equal to 0.76 UFL. Thus, energy requirements are equal to 1.29 UFL (0.69 UFL maintenance, plus 0.60 UFL milk production). These data have been confirmed by several studies conducted on grazing Cilentana goats (D’Urso et al., 2008; Tudisco et al., 2010, 2014).

These values are in accordance to the equation for small ruminants reported by NRC (I = 0.04 × A × Z(1.7-Z)) where *A* stands for ‘standard reference weight’ defined as the weight of nonpregnant animal when it reaches mature size and a condition score in the middle of the range used and *Z* stands for ‘relative size’ which is the ratio of the normal weight of an immature animal at a given age to its mature weight as defined by A (Nutrient Requirements of Small Ruminants sheep, goats, cervids, and new world camelids, 2007). Thus, if we consider an average weight of 50 kg, which is both the mature size of Cilentana female goat (A) and goats’ current weight, we are considering (A × Z); the equation predicts an intake of 1.4 kg of pasture DM.

When fodder is administered, the main hays used are oat or alfalfa ad libitum (Tudisco et al. 2010, 2012).

Animals’ diet is supplemented with concentrate at a rate of 200–300 and 400 g/head/day, respectively, 45–30 and 15 days before the presumed date of kidding; successively, the amount of concentrate will be calculated according to the productive capacity of the flock and the vegetative state of the pasture.

## Cilentana breed production performance

Cilentana is considered a dual-purpose breed; thus, its productions are related both to meat and to milk for processing dairy products.

From a reproductive point of view, it has a seasonal polyestral sexual cycle. With the aim of getting kids for the Easter (early spring), the farmers regulate the sex promiscuity. Cilentana kids reach the puberty at 7–8 months of age and the first kidding is around 1 year; the duration of gestation varies between 145.3 and 150.6 days. The fertility ranges between 90 and 95% and prolificity rates are more than 185% (Noè et al., 2005).

Campania region has a considerable heritage in terms of typical and valuable productions which imply a great degree of entrepreneurial skills on the part of the producers themselves. The protection of the peculiarities of those productions is considered a strong lever for the marketing and affirmation of the products on the market (Infascelli et al., 2021).

### Milk production

Lactation lasts about 7 months, generally from February to August due to the seasonal polyestral sexual cycle. When diet is well balanced, as above described according to several studies (D’urso et al., 2008; Tudisco et al., 2010, 2014), average milk yield is about 1396 g/day with a protein content of 3.59%, fat 4.54% and lactose 4.60%. In these works, the characteristics of milk were compared according to the differing animals feeding conditions. The values mentioned relate to the results obtained by goats fed on pasture, since this type of feeding system is the most common in this area.

With regard to milk fatty acid profile, the averages obtained from the same works are reported in Table 1 for the content in saturated fatty acids (SFA), monounsaturated fatty acids (MUFA), polyunsaturated fatty acids (PUFA) and total CLA.

**Table 1:** Mean value of milk yield, chemical composition and MUFA, PUFA and SFA content from the three different studies

	D’Urso et al. 2008	Tudisco et al. 2010	Tudisco et al. 2014	Mean value	SD
Number of animals	15	15	15	-	-
Yield (g/day)	1337	1432	1420	1396	± 51.73
Fat (%)	4.60	4.59	4.45	4.54	± 0.08
Protein (%)	3.58	3.57	3.62	3.59	± 0.02
Lactose (%)	4.59	4.58	4.65	4.60	± 0.03
SFA (g/100 g of fat)	59.64	58.94	73.42	64	± 8.16
MUFA (g/100 g of fat)	20.32	23.01	22.92	22.08	± 1.52
PUFA (g/100 g of fat)	4.00	4.52	3.47	3.99	± 0.52
CLA (g/100 g of fat)	0.84	0.87	0.87	0.86	± 0.01

*SFA*, saturated fatty acids; *MUFA*, monounsaturated fatty acids; *PUFA*, polyunsaturated fatty acids; *CLA*, conjugated linoleic acid; *SD*, standard deviation

As shown in Table 2, Cilentana milk yield, chemical composition and fatty acid profile differ from other breeds’ one. In particular, Cilentana goats’ milk showed higher value of fat and a fatty acid profile with a lower percentage of SFA, while having a lower milk yield. The works reported in Table 2 were all conducted on grazing goats with similar diet composition; thus, the different results are mainly due to the breed’s characteristics

**Table 2:** Comparison of Cilentana, Saanen and Alpine goats mean values of milk yield, chemical composition and MUFA, PUFA and SFA.

	Cilentana goats (mean values from Table 1)	Alpine goats (Lopez et al. 2019)	Saanen goats (Currò et al. 2019)
Yield (g/day)	1396	2550	1730
Fat (%)	4.54	3.2	3.61
Protein (%)	3.59	3.2	3.20
Lactose (g/day)	4.60	4.4	4.34
SFA (g/100 g of fat)	64	69.8	70.35
MUFA (g/100 g of fat)	22.08	21.9	24.03
PUFA (g/100 g of fat)	3.99	3.6	5.31
CLA (g/100 g of fat)	0.86	N.M	0.92

*SFA*, saturated fatty acids; *MUFA*, monounsaturated fatty acids; *PUFA*, polyunsaturated fatty acids; *CLA*, conjugated linoleic acid; *N.M.*, not measured

### Cheese production

**Fig. 6:**
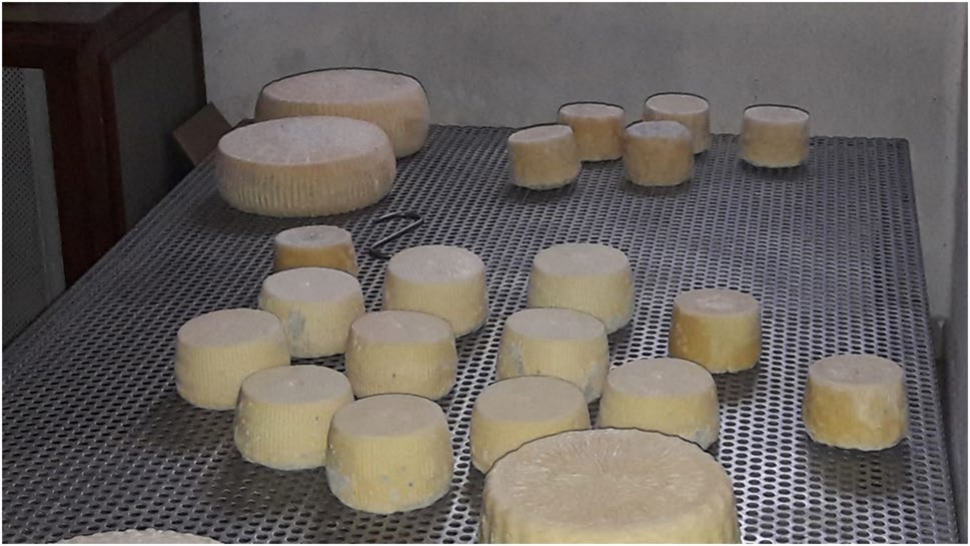
Typical Cacioricotta cheese from Cilentana goat

Cacioricotta is a typical goat cheese made from fresh milk whose name derives from the peculiar process of milk coagulation combining ricotta and cheese manufacturing (Fig. 6). It originates from the Cilento area, near Salerno, where it has been produced and marketed for years. It is cylindrical and pale yellow or darker in colour and has an intense flavour depending on the time taken for ripening. Initially, the milk is heated to 85–90 °C for 15–20' in order to get most of the whey proteins precipitate. It is then left to cool naturally up to about 37 °C, and then kid rennet is added. The curd is broken and then reassembled in the ‘fuscelle’, typical wicker baskets which allow the whey to drain for 24 h. The particular combination of heat and curd determines the coagulation of the milk proteins, that is the ‘cacium’ (cheese), and of the whey which becomes ricotta. The product is eaten fresh or seasoned. The ripening makes it hard, compact and crumbly. Currently, the ‘Cacioricotta del Cilento’ has been included among the Italian Traditional Agri-food Products (PAT) and is a Slow Food presidium, supported by the Cilento, Vallo di Diano and Alburni National Park (Regione Campania: Assessorato all’agricoltura, 2020, http://www.agricoltura.regione.campania.it/tipici/tradizionali/cacioricotta-cilento.htm).

### Meat production

The goat meat market mainly concerns the sale of kids, especially during Easter. Kids are generally slaughtered very young, at the age of 2–3 months.

In a traditional local festival, however, adult goat meat is also consumed, mainly from Cilentana breed. The ‘Frecagnola festival’ takes place in Cannalonga (SA) on the Saturday preceding the second Sunday of September, and over time, it has been characterized by the buying and selling of livestock. The dish typically consumed in this festival is the traditional boiled goat ‘la crapa vudduta’.

With regard to the characteristics of the meat, few studies have been done on Cilentana goat meat. In the work of Cutrignelli et al. (2007), the production performances of Cilentana kids were considered in function of two different feeding systems administered to the mothers (stable vs pasture). Birth weight was on average (between the two groups) of 3.93 kg with the daily increase of 178.6 g from day 1 to day 25 and of 132.65 g considering from day 1 to day 60 with a final weight of about 12 kg. Cilentana kid carcass performances (Tudisco et al., 2015) compared to Saanen and Alpine kids are reported in Table 3. Even if the slaughtering weight was different, Cilenata kids’ carcass shows similar characteristics when compared to other breeds.

**Table 3:** Cilentana and Saanen kid performances

	Tudisco et al. (2015)	Cimmino et al. (2018)	Andrighetto et al. (1994)
Breed	Cilentana	Saanen	Alpine
Age at slaughtering (days)	60	146.8	-
Slaughtering weight (kg)	12.5	17.9	13.4
Hot carcass weight (kg)	7.2	10.0	8.2
Cold carcass weight (kg)	6.9	9.7	7.7
Hot dressing (%)	57.6 ± 0.3	55.8	65.1
Cold dressing (%)	55.2 ± 0.4	54.6	63.0

Goat carcass is generally smaller and has a lower fat cover compared to sheep (Webb et al., 2005). The fat development also occurs later, and contrary to sheep, the fat is deposited mainly at the perivisceral level rather than in the carcass (Babiker et al., 1989).

Due to the more favourable nutritional characteristics compared to the rest of red meat, the consumption of goat meat is increasing worldwide (Mazhangara et al., 2019). From a nutritional point of view, goat meat, both adults and kids, besides being a good source of animal protein, distinguishes itself for total fat, saturated fatty acids and cholesterol content which makes it a healthier product (Ivanović et al., 2016).

## The Common Agricultural Policy post 2023

The new Common Agricultural Policy (Cap) in the aims of both the European Union (EU) Commission and Parliament will have a role to contribute to the greater environmental ambition, through a wide range of interventions aimed at specific needs and tangible results in relation to the objectives at EU level, while at the same time contributing to the Green Deal (De Castro et al., 2011, 2012).

According to the recommendations of the Commission, each Member State will have to demonstrate the greatest environmental ambition with its National Strategic Plan (NSP), which it will have to update with the relevant elements established by future legislation on climate and environment.

To this end, Member States will have the following tools at their disposal:

- Enhanced conditionality: CAP beneficiaries will have to comply with a stronger set of mandatory requirements including good agricultural and environmental conditions (GAEC), mandatory management criteria (CGO) and the criteria for greening.
- Ecological schemes: at least 25% of the envelope for direct payments will be allocated to eco-schemes, providing incentives for practices and agriculture in favour of the climate and the environment (such as organic farming, agroecology, agriculture carbon) as well as the improvement of animal welfare.
- Rural development: at least 35% of the Fears budget will be devoted to measures to support climate, biodiversity, the environment and animal welfare.
- Climate and biodiversity: 40% of the CAP budget will have to be allocated to spending on climate and the environment, with the general commitment to dedicate 10% of the EU budget to biodiversity objectives by the end of the period.

Key elements of this architecture will be either the enhanced compliance or the social compliance that would play a crucial role for an economic sustainability of Cilentana breed and mountain area more generally.

Specifically, the cross-compliance system applies to all beneficiaries who receive direct payments and related annual payments for agri-climate-environment commitments, natural constraints and specific territorial handicaps envisaged by rural development. The new system includes a list of Statutory Management Requirement (SMR) and rules for the maintenance of land in good agricultural and environmental condition (9 GAEC standards) with a greater focus on environmental and climatic challenges, including the current criteria for the greening (enhanced conditionality).

SMRs are requirements established by a list of legal acts in force in the EU and, in the case of directives, as implemented by the Member States.

The GAEC framework includes nine mandatory practices (two more than the previous legislation) to address the challenges of climate change mitigation, water protection and management, soil quality and biodiversity.

Failure to comply with cross-compliance rules implies an effective and proportionate system of administrative sanctions for non-compliance, which is included in the national strategic plan.

The co-legislators and the Commission, in order to contribute to the development of socially sustainable agriculture, have established a new system aimed at ensuring respect for workers’ rights, employment and social standards (De Castro et al., 2014).

The mechanism conditions the granting of direct payments and payments for commitments relating to the environment and climate and for other commitments relating to management, for natural or other constraints and mandatory payments for specific disadvantages, to the respect by the beneficiary of legal rules relating to the working and employment conditions of agricultural workers, including health and safety at work.

It will be up to the competent national authorities to carry out checks and to transfer a list of the infringements found to the competent paying agencies. The latter will therefore have the task of imposing on the beneficiary a sanction proportionate to the seriousness of the acts, effective and dissuasive for farmers who do not comply with these requirements.

The new system will have to be compulsorily implemented for all farmers in all Member States, starting from 2025. Agricultural advisory services can be used, including at sectoral level, to improve the sustainable management of farms, economically and environmentally, and social, and then identify the necessary improvements to be made.

### The second pillar of the CAP

In line with the provisions of the new implementation model, rural development will also be a unique part of the NSP, which may include programs established at the regional level ensuring consistency and consistency with the elements of the NSP established at national level.

The new programming includes eight types of intervention:

- Environmental and climate commitments and other management commitments
- Natural or other specific territorial constraints
- Specific territorial disadvantages deriving from certain mandatory requirements
- Investments, including investments in irrigation
- Establishment of young farmers and new farmers, and the start-up of rural businesses
- Risk management tools
- Cooperation
- Exchange of knowledge and dissemination of information

Points a, b, c, e, d and h would play a crucial role aiming to increase the economic sustainability of farmers in mountain and rural area; in this view, Cilentana breed would benefit of new second pillar framework, provided that the regional authorities responsible for planning rural development policies really have the objective of supporting this type of farmers.

For example, in areas with natural or other specific constraints, in National Strategic Plans (NSPs), specific support could be implemented aimed to overcome difficulty in cultivating.

The support is paid through payments granted to active farmers whose holding area is located in designated nitrate vulnerable directive (NVD) areas, including mountain and island areas. Payments are granted only in order to compensate, in whole or in part, the beneficiaries for the additional costs and loss of earnings due to natural or other specific territorial constraints in the area concerned.

The current definition of the criteria for the delimitation of these areas remains.

Among all, the measures ‘conversion of arable land to meadows and pastures’ and ‘management of permanent meadows and pastures’ appear to be very promising.

In the former case, the intervention involves the conversion of alternated arable land to more extensive forms of use that do not involve working the land and the use of plant protection products and herbicides.

In order to improve the environmental performance envisaged by the intervention, it is possible to strengthen the commitments envisaged with those of some other agro-climatic-environmental interventions. The intervention can be implemented by single or associated farmers, farms of public bodies, collective entities in the context of the cooperation intervention and other land managers, including collective properties, limited to agricultural areas; payments per hectare of utilized agricultural area (UAA) are granted for a period of 5 years and differentiated support could be envisaged according to the altitude zones (plains, hills, mountains).

In the latter, the intervention is aimed at the sustainable management of permanent meadows and pastures, as areas with a high proportion of semi-natural vegetation, and therefore considered agricultural areas of high naturalistic value (HVN). Sustainable management promotes biodiversity, limits soil erosion and degradation processes, eliminates the contribution of chemical and mineral fertilizers and pesticides and improves, within the LULUCF sector (Land Use, Land Use Change, Forestry), the absorption of CO_2_ and adaptability to extreme weather events.

The intervention is divided into three types of operations (sustainable management of permanent lawns, sustainable management of meadows-pastures, sustainable management of permanent pastures) and can be implemented by single or associated farmers, farms of public bodies, single or associated entities, of a public or private nature, managers of the areas involved in the commitment and collective entities in the context of the cooperation intervention. Per hectare payments of UAA are granted for a period of 5 years.

## Conclusions and future perspectives

The safeguard of indigenous breeds aims at the protection of biodiversity and the conservation of local activities that have a historical tradition. The presence of these farms preserves also the territory itself and guarantees a source of income for the communities in otherwise abandoned territories. Cilentana goat is well adapted to its territory of origin and it produces high-quality cheese and meat. The possibility of working on the characterization of these animals’ meat is certainly among the future prospects, since few studies have been done on the matter.

The organoleptic and nutritional characteristics of a product linked it to its territory of origin are aspects to be considered in order to enhance these productions and communicate their real value to the consumers. Through the text, we introduced a brief representation of the objectives and the new intervention structure of the post-2023 CAP. The analysis and objective of this representation was to introduce the strategic importance of public community intervention which, compared to the past, even more recently, it has introduced profound innovations in both objectives and tools. This novelty represents a crucial opportunity, to be built and seized, to ensure an economic sustainability also for the agricultural activity of the inland areas and for those farmers who dedicate their business to extensive farms such as the one represented by Cilentana. Failing to fully implement the potentialities envisaged by the CAP intervention could represent an insurmountable obstacle for the survival of extensive pastures in inland areas, that is, heavily impacting the animal and vegetable biodiversity of these areas which represent more than half of the Italian agricultural surface.

## Data Availability

Not applicable*.*
